# 4386 Age-related Changes in the Functional Connectivity within the Default Mode Network

**DOI:** 10.1017/cts.2020.166

**Published:** 2020-07-29

**Authors:** Cassandra Leonardo, Crystal G Franklin, Peter T Fox

**Affiliations:** 1 University of Health Science Center at San Antonio

## Abstract

OBJECTIVES/GOALS: To evaluate whether the default mode network experiences age-related changes in functional connectivity and to identify these patterns of progression across seven decades of life. The overall goal is to evaluate whether quantifying these functional changes can serve as potential neurobiomarkers of aging for further quantitative genetic analyses. METHODS/STUDY POPULATION: Scans were performed at the RII on a 3T Siemens Trio scanner with an 8-channel head coil. Whole-brain, rsfMR imaging was performed using a gradient-echo EPI sequence sensitive to the BOLD effect (TE/TR = 30/3000 ms; flip angle = 90°; isotropic 1.72 mm^2^). Subjects were instructed to lie in dimmed light with their eyes open and try not to fall asleep. Image analysis was performed with FMRIB’s Software Library tools (www.fmrib.ox.ac.uk/fsl). Preprocessing of resting state data includes motion correction, brain extraction, spatial smoothing, and high-pass temporal filtering. Time series data was extracted from 9 DMN ROIs using FSL’s Featquery tool with 6mm radius spherical ROI masks created in Mango. After extraction, DMN connectivity was assess using structural equation modeling implemented in Amos 22.0 (IBM, Inc.). RESULTS/ANTICIPATED RESULTS: The exploratory SEM (eSEM) default mode network (DMN) model used consists of 9 regions of interest and 13 functional connectivity paths. The eSEM DMN model exhibited exceptional model fit to a primary resting state data set of 1169 subjects from the Genetics of Brain Structure project (1R01MH078111-01, David Glahn PI) with an RMSEA of 0.037. This model also had excellent model fit in 7 cohorts that were grouped by decade age (10s – RMSEA: 0.058, 20s – 0.051, 30s – 0.045, 40s – 0.048, 50s – 0.043, 60s – 0.035, 70s – 0.037). Analysis of the decade group-wise path coefficients identified 7 of the 13 paths (pC -> LMTG, pC -> PCC, PCC -> MPFG, PCC -> vACC, MPFG -> vACC, LIPL -> RIPL, LMTG -> RMTG) significantly negatively correlated with age-related changes. As early as the 1^st^ decade of life, the functional connectivity within the DMN decreases. DISCUSSION/SIGNIFICANCE OF IMPACT: The DMN experiences progressive age-related decreases in connectivity, beginning in the first decade of life. Our results suggest that DMN path coefficients can serve as biomarkers of cognitive aging, which can then be used as quantitative traits for genetic analyses to identify genes associated with normal aging and age-related cognitive diseases.

